# A resurgence of e-cigarette use among adolescents and young adults late in the COVID-19 pandemic

**DOI:** 10.1371/journal.pone.0282894

**Published:** 2023-03-29

**Authors:** Jennifer M. Kreslake, Katie M. O’Connor, Michael Liu, Donna M. Vallone, Elizabeth Hair

**Affiliations:** 1 Truth Initiative Schroeder Institute, Washington, DC, United States of America; 2 Johns Hopkins Bloomberg School of Public Health, Baltimore, MD, United States of America; 3 New York University School of Global Public Health, New York, NY, United States of America; Frederick National Laboratory for Cancer Research, UNITED STATES

## Abstract

**Background:**

Early in the COVID-19 pandemic, e-cigarette use significantly declined among young people due, in part, to losing access through social sources. As the pandemic progressed, adolescents and young adults gained opportunities to resume contact with peers. This study sought to determine whether e-cigarette use has returned to pre-pandemic levels among adolescents and young adults.

**Methods:**

Data were drawn from a cross-sectional weekly survey of adolescents (aged 15–17) and young adults (aged 18–24) (N = 37,331). Logistic regression analyses measured odds of past 30-day e-cigarette use among respondents surveyed (a) late in the pandemic (April 2021—April 2022) compared to early in the pandemic (March–July 2020) and (b) late in the pandemic (August–December 2021) compared to prior to the pandemic (August–December 2019).

**Results:**

The odds of current e-cigarette use were significantly higher later in the COVID-19 pandemic (April 2021–April 2022), compared to its initial months (March–July 2020) (OR:1.27, 95% CI: 1.17–1.38; p = 0.001). There was no significant difference in the odds of e-cigarette use for youth and younger adults late in the pandemic (August–December 2021) compared to the same time period prior to the pandemic (August–December 2019), but odds were greater for young adults aged 21 years or older (OR:1.16; 95% CI: 1.01–1.32; p = 0.030).

**Conclusions:**

E-cigarette use has returned to pre-pandemic levels among adolescents and young adults. Young adults over age 21 are more likely to use e-cigarettes than young adults of the same age surveyed prior to the pandemic. Findings have implications for targeted e-cigarette prevention and cessation efforts.

## Background

Between 2015 and 2019, e-cigarette use had been rising rapidly among youth and young adults in the United States. In 2015, 16.0% of US high school students [1] and 5.2% of young adults (aged 18–25 years) reported current e-cigarette use [2]. Comparatively, in 2019, 27.5% of high school students [3] and 9.3% of young adults reported current e-cigarette use [4]. Since COVID-19 was declared a pandemic in March 2020 [5], multiple public health measures have been introduced to contain the spread of the virus in the United States. Previous research demonstrated a significant drop in e-cigarette use among young people during the first several months of the COVID-19 pandemic [6]. Declines in e-cigarette use were attributed to reduced access to retail stores and social sources, an unintended consequence of nationwide lockdowns that prompted temporary closures of schools and nonessential businesses. While these restrictions to reduce COVID-19 exposure appeared to curb e-cigarette use among many young people early in the pandemic, it is currently unknown whether the decline in prevalence has been sustained as restrictions have been lifted.

Our previous research demonstrated that a decline in e-cigarette use could be observed among those under aged 21 during the earliest months of the pandemic (March-July 2020) [6]. A more recent study by Bennett et al (2023) conducted one year into the pandemic (April-June 2021) found that e-cigarette users reported varying accounts of whether they changed their e-cigarette use behaviors; over one-third (38%) reported increasing their e-cigarette use, slightly more than one-quarter (29%) reported decreasing use, and one-third (33%) sustained the same level of use compared to prior to the COVID-19 pandemic [7]. However, these findings relied upon a retrospective, self-reported measure comparing one’s frequency of current use to “before the COVID-19 pandemic,” which could be subject to recall bias as well as different interpretations of the pre-pandemic time period by the respondent. While the Bennett et al findings suggest that various responses to the COVID-19 pandemic may contribute to sustained or increased e-cigarette use, these results cannot be used to estimate changes in prevalence among the general population before and over the course of the pandemic. Research is needed to establish whether rates of e-cigarette use have changed significantly among youth and young adults using prevalence measures taken prior to, and compared to those taken throughout, the COVID-19 pandemic.

Prior to COVID-19, peers represented the primary source of obtaining tobacco products among adolescents and young adults. A study conducted by Liu et al. (2019) found that youth were significantly more likely to obtain e-cigarettes and other tobacco products from social sources [8], which is consistent with earlier studies [9, 10]. It remains unclear as to how sources of e-cigarettes among young people may have changed over the course of the COVID-19 pandemic.

This study will examine changes in prevalence of e-cigarette use among adolescents and young adults in the United States over the course of COVID-19. First, we will determine whether respondents surveyed later in the COVID-19 pandemic (April 21 2021—April 26 2022) are more likely to report current e-cigarette use compared to those surveyed during the initial months of the pandemic (March 18—August 4 2020). Secondly, we will determine whether respondents surveyed later in the COVID-19 pandemic (July 28—December 28 2021) are as likely to report current e-cigarette use compared to those surveyed during a comparable time span prior to COVID-19 (July 31—December 31 2019).

## Methods

The study used data from a continuous cross-sectional online panel survey of adolescents (aged 15–17) and young adults (aged 18–24 years). A total of 37,331 respondents were surveyed between July 31, 2019 and April 26, 2022. Approximately 280 unique respondents were sampled per week using sampling quotas to achieve equal proportions of each age group and sex. Data were weighted to be nationally representative using demographic characteristics according to the US Census. The study received approval from the Advarra Institutional Review Board.

Data were aggregated by month to determine the monthly prevalence of e-cigarette use in the sample. Logistic regression models were used to determine whether an observed change in past 30-day e-cigarette was significantly greater among adolescents and young adults in later months of the study period. Each respondent was surveyed once during the study period, thus the pre-post comparisons between responses were unpaired and aggregated for each time period.

Based on previous research demonstrating differences in COVID-19-related trends in e-cigarette use by age, models were stratified by age group (15–17 years, 18–20 years, and 21–24 years) [6]. Current e-cigarette use, defined as use on at least 1 day during the past 30 days, was the outcome. Other covariates included race/ethnicity (non-Hispanic white, non-Hispanic Black, non-Hispanic and other race, or Hispanic), a validated measure of subjective financial situation to serve as a proxy for socioeconomic status, sensation-seeking score, and sex [11]. We used a fixed effect for respondent’s state of residence to control for variations in state-level e-cigarette policies and other interventions (e.g., age restrictions, anti-vaping mass media campaigns) and COVID-19 responses (e.g., vaccination roll-out schedules and uptake, school closures).

## Results

Approximately one-quarter (27.8%) of the sample surveyed during the study period (August 2019 to April 2022) were between the ages of 15 and 17 years, slightly less than one-third (31.1%) were aged 18 to 20, and the remainder (41.1%) were aged 21 to 24. Half (50.7%) of the sample was male. Slightly more than half (57.2%) were white and non-Hispanic. One-quarter (25.0%) identified as having Hispanic ethnicity. Black non-Hispanic and other non-Hispanic respondents made up smaller proportions of respondents (12.9% and 7.1%, respectively). The majority (59.4%) reported that their financial circumstances met their basic needs with a little left over, or to live comfortably. One-quarter (27.3%) of the sample, aggregated over the course of two years, were current e-cigarette users (Table 1).

**Table 1:** Survey respondent characteristics, July 2019 –May 2022, United States (N = 37,331).

	Full Sample	Model 1: Early or Late Pandemic^a^ (n = 20,641)	Model 2: Pre- or Late Pandemic^b^ (n = 11,397)
	n (weighted %)	n (weighted %)	n (weighted %)
Age category			
15–17	10347 (27.8)	5903 (28.8)	3124 (27.6)
18–20	11730 (31.1)	6286 (30.1)	3612 (31.2)
21–24	15254 (41.1)	8452 (41.1)	4661 (41.2)
Sex			
Male	18940 (50.7)	10457 (51.2)	5746 (51.2)
Female	18391 (49.3)	10184 (48.9)	5651 (48.8)
Race/ethnicity			
White, non-Hispanic	19463 (57.2)	10377 (54.1)	5792 (56.8)
Black, non-Hispanic	4557 (12.9)	2555 (12.9)	1398 (12.2)
Hispanic	8833 (25.0)	4918 (24.0)	2650 (22.0)
Other, non-Hispanic	2493 (7.1)	1763 (9.1)	964 (9.0)
Perceived financial situation^c^			
Low	15235 (40.6)	8253 (39.8)	4830 (42.1)
High	22096 (59.4)	12388 (60.2)	6567 (57.9)
Sensation-seeking score (mean, SE)	3.27 (0.004)	3.26 (0.01)	3.26 (0.01)
Current e-cigarette use (past 30-day)	10176 (27.3)	5553 (26.9)	3203 (28.2)

^a^Respondents surveyed March–July 2020 or April 2021 –April 2022

^b^Respondents surveyed August–December 2019 or August–December 2021

^c^Lower perceived financial circumstances do not meet basic needs or just met needs with nothing left over. Higher perceived financial circumstances meet basic needs with a little left over or allow the respondent to live comfortably.

For the first analyses testing the odds of e-cigarette use late in the COVID-19 pandemic compared to the pandemic’s early months, significant differences in e-cigarette use were found according to the phase of the pandemic in which respondents were interviewed (Table 2). Respondents surveyed from April 2021 to April 2022 had significantly higher odds of e-cigarette use compared to those surveyed early in the pandemic (March–July 2020) (OR: 1.27, 95% CI: 1.17–1.38, p<0.001). This finding remained significant in models stratified by each age group. For adolescents aged 15–17, the odds of e-cigarette use were 1.22 (95% CI: 1.01–1.49; p = 0.043) late in the pandemic compared to its early stages; for those aged 18–20 years, the odds were 1.41 (95% CI: 1.21–1.64, p<0.001); and for those aged 21–24, the odds were 1.18 (95% CI: 1.05–1.33, p = 0.007) (Table 2).

**Table 2:** Odds of past 30-day e-cigarette use late in the COVID-19 pandemic (April 2021– April 2022) compared to early in the pandemic (March–July 2020) among youth and young adults in the United States, age-adjusted or stratified by age group.

	All ages (15–24 years) n = 19,600	Ages 15–17 n = 5,529	Ages 18–20 n = 5,947	Ages 21–24 n = 8,124
	OR (95% CI)	OR (95% CI)	OR (95% CI)	OR (95% CI)
Survey period^a^				
Early COVID-19 pandemic	REF	REF	REF	REF
Late COVID-19 pandemic	1.27 (1.17, 1.38)	1.22 (1.01, 1.49)	1.41 (1.21, 1.64)	1.18 (1.05, 1.33)
Age category				
15–17	0.38 (0.35, 0.42)			
18–20	0.74 (0.69, 0.80)			
21–24	REF			
Race/ethnicity				
White, non-Hispanic	REF	REF	REF	REF
Black, non-Hispanic	0.59 (0.53, 0.66)	0.53 (0.40, 0.71)	0.56 (0.46, 0.69)	0.61 (0.52, 0.72)
Hispanic	0.85 (0.78, 0.92)	0.88 (0.73, 1.06)	0.70 (0.60, 0.82)	0.91 (0.80, 1.02)
Other, non-Hispanic	0.78 (0.69, 0.89)	0.77 (0.59, 1.02)	0.75 (0.60, 0.94)	0.82 (0.68, 0.98)
Perceived financial circumstances^b^				
Low	1.42 (1.33, 1.52)	1.99 (1.70, 2.33)	1.58 (1.40, 1.78)	1.19 (1.08, 1.31)
High	REF	REF	REF	REF
Sensation seeking	1.73 (1.66, 1.81)	1.84 (1.66. 2.03)	1.63 (1.50, 1.77)	1.76 (1.65, 1.88)
Sex				
Male	0.99 (0.93, 1.06)	0.82 (0.71, 0.95)	0.86 (0.77, 0.97)	1.16 (1.05, 1.27)
Female	REF	REF	REF	REF
State of residence				
Alaska	0.36 (0.18, 0.72)	0.91 (0.27, 3.02)	0.21 (0.06, 0.80)	0.35 (0.13, 0.97)
Arizona	0.92 (0.67, 1.27)	1.07 (0.56, 2.04)	0.63 (0.35, 1.13)	1.06 (0.66, 1.69)
Arkansas	0.88 (0.62, 1.25)	0.49 (0.19, 1.25)	0.61 (0.31, 1.19)	1.31 (0.81, 2.13)
California	0.80 (0.62, 1.02)	0.62 (0.37, 1.05)	0.75 (0.48, 1.15)	0.90 (0.63, 1.30)
Colorado	0.93 (0.66, 1.31)	0.86 (0.43, 1.70)	0.88 (0.47, 1.63)	0.96 (0.58, 1.60)
Connecticut	0.92 (0.64, 1.33)	0.57 (0.24, 1.33)	1.16 (0.62, 2.16)	0.92 (0.53, 1.60)
Delaware	0.58 (0.33, 1.03)	0.20 (0.05, 0.84)	0.58 (0.23, 1.47)	0.88 (0.37, 2.07)
District of Columbia	0.66 (0.31, 1.44)	0.70 (0.09, 5.55)	1.02 (0.20, 5.21)	0.60 (0.23, 1.55)
Florida	0.80 (0.62, 1.04)	0.69 (0.40, 1.18)	0.79 (0.51, 1.23)	0.86 (0.59, 1.25)
Georgia	0.92 (0.70, 1.21)	0.63 (0.34, 1.14)	0.92 (0.58, 1.47)	1.07 (0.72, 1.61)
Hawaii	1.02 (0.54, 1.94)	1.18 (0.29, 4.77)	0.85 (0.26, 2.81)	1.08 (0.44, 2.67)
Idaho	0.63 (0.35, 1.11)	0.30 (0.06, 1.37)	0.74 (0.26, 2.04)	0.77 (0.34, 1.74)
Illinois	0.85 (0.64, 1.13)	0.60 (0.32, 1.13)	0.80 (0.49, 1.30)	1.02 (0.68, 1.53)
Indiana	0.94 (0.69, 1.30)	1.01 (0.53, 1.92)	0.81 (0.47, 1.41)	0.98 (0.62, 1.57)
Iowa	0.78 (0.50, 1.21)	0.77 (0.31, 1.91)	0.84 (0.44, 1.63)	0.67 (0.32, 1.42)
Kansas	0.77 (0.50, 1.19)	1.09 (0.52, 2.31)	0.88 (0.39, 1.98)	0.59 (0.30, 1.13)
Kentucky	0.94 (0.67, 1.31)	0.84 (0.40, 1.75)	0.97 (0.55, 1.71)	0.92 (0.56, 1.51)
Louisiana	0.94 (0.67, 1.32)	0.89 (0.42, 1.91)	1.0 (0.59, 1.80)	0.88 (0.53, 1.43)
Maine	0.40 (0.21, 0.76)	0.14 (0.02, 1.23)	0.73 (0.30, 1.80)	0.27 (0.09, 0.81)
Maryland	0.79 (0.57, 1.09)	0.56 (0.25, 1.25)	0.57 (0.32, 1.03)	1.06 (0.68, 1.69)
Massachusetts	0.98 (0.70, 1.36)	0.74 (0.36, 1.52)	1.06 (0.60, 1.87)	1.02 (0.62, 1.70)
Michigan	1.02 (0.77, 1.36)	0.93 (0.52, 1.66)	0.97 (0.60, 1.60)	1.07 (0.70, 1.63)
Minnesota	0.88 (0.63, 1.24)	0.68 (0.33, 1.42)	1.07 (0.60, 1.94)	0.85 (0.52, 1.42)
Mississippi	0.88 (0.60, 1.31)	1.14 (0.48, 2.71)	0.77 (0.42, 1.46)	0.95 (0.52, 1.71)
Missouri	0.97 (0.70, 1.35)	0.98 (0.51, 1.88)	1.14 (0.66, 1.97)	0.81 (0.50, 1.33)
Montana	0.71 (0.34, 1.51)	0.37 (0.66, 2.06)	0.37 (0.07, 1.85)	1.38 (0.47, 4.10)
Nebraska	2.51 (1.62, 3.87)	0.68 (0.25, 1.89)	0.97 (0.43, 2.18)	8.10 (3.71, 17.67)
Nevada	1.24 (0.81, 1.90)	1.20 (0.55, 2.60)	1.63 (0.72, 3.69)	1.08 (0.58, 2.01)
New Hampshire	0.72 (0.38, 1.38)		1.35 (0.41, 4.40)	0.89 (0.37, 2.11)
New Jersey	0.87 (0.64, 1.19)	0.70 (0.36, 1.37)	1.01 (0.60, 1.71)	0.87 (0.55, 1.36)
New Mexico	0.74 (0.39, 1.40)	0.75 (0.21, 2.64)	0.54 (0.17, 1.70)	0.88 (0.34, 2.29)
New York	0.79 (0.61, 1.02)	0.70 (0.40, 1.21)	0.65 (0.42, 1.03)	0.93 (0.64, 1.34)
North Carolina	0.87 (0.66, 1.17)	0.88 (0.48, 1.63)	0.64 (0.38, 1.06)	1.07 (0.70, 1.64)
North Dakota	0.86 (0.33, 2.27)	0.59 (0.78, 4.50)	0.97 (0.19, 4.83)	0.92 (0.19, 4.44)
Ohio	0.74 (0.56, 0.99)	0.50 (0.26, 0.93)	0.69 (0.43, 1.11)	0.92 (0.61, 1.39)
Oklahoma	0.76 (0.53, 1.08)	0.14 (0.03, 0.61)	0.73 (0.38, 1.39)	1.08 (0.66, 1.79)
Oregon	1.08 (0.73, 1.60)	0.64 (0.25, 1.67)	1.09 (0.54, 2.22)	1.31 (0.75, 2.28)
Pennsylvania	0.86 (0.65, 1.13)	0.77 (0.44, 1.39)	1.02 (0.64, 1.63)	0.77 (0.51, 1.17)
Rhode Island	0.74 (0.38, 1.41)	0.55 (0.11, 2.82)	1.06 (0.35, 3.22)	0.70 (0.28, 1.77)
South Carolina	0.93 (0.66, 1.30)	0.77 (0.37, 1.63)	0.74 (0.40, 1.38)	1.13 (0.70, 1.85)
South Dakota	1.01 (0.46, 2.22)	2.01 (0.63, 6.34)	0.22 (0.03, 1.78)	1.16 (0.34, 3.89)
Tennessee	0.88 (0.65, 1.20)	0.66 (0.34, 1.27)	1.20 (0.72, 2.03)	0.82 (0.52, 1.27)
Texas	0.84 (0.65, 1.09)	0.60 (0.35, 1.02)	0.81 (0.52, 1.24)	1.02 (0.71, 1.48)
Utah	0.68 (0.46, 1.00)	0.36 (0.12, 1.04)	0.68 (0.31, 1.47)	0.78 (0.46, 1.31)
Vermont	0.99 (0.42, 2.34)	0.94, (0.06, 12.50)	0.79 (0.22, 2.87)	1.23 (0.34, 4.76)
Virginia	0.72 (0.53, 0.98)	0.43 (0.20, 0.92)	0.79 (0.46, 1.33)	0.80 (0.51, 1.26)
Washington	0.84 (0.59, 1.17)	0.64 (0.32, 1.27)	0.82 (0.45, 1.50)	0.96 (0.58, 1.58)
West Virginia	0.81 (0.50, 1.32)	1.16 (0.46, 2.93)	0.51 (0.22, 1.90)	0.88 (0.43, 1.83)
Wisconsin	0.76 (0.53, 1.09)	0.54 (0.25, 1.15)	0.94 (0.52, 1.69)	0.75 (0.43, 1.32)
Wyoming	0.74 (0.32, 1.69)	0.93 (0.22, 3.96)	0.33 (0.69, 1.56)	1.05 (0.29, 3.79)

^a^Respondents surveyed March–July 2020 were classified in the “Early COVID-19 pandemic” category, and those surveyed April 2021 –April 2022 were classified in the “Late COVID-19 pandemic” category.

^b^Lower perceived financial circumstances do not meet basic needs or just met needs with nothing left over. Higher perceived financial circumstances meet basic needs with a little left over or allow the respondent to live comfortably.

For the second analyses testing the odds of e-cigarette use late in the COVID-19 pandemic compared to a similar time span before the pandemic, there was no significant difference in e-cigarette use during the late-COVID-19 period (August–December 2021) compared to the same time span prior to the pandemic (August–December 2019) (Table 3). These findings were consistent in models stratified among underage respondents (aged 15–17 and 18–20 years). However, the odds of e-cigarette use among young adults aged 21–24 was significantly higher during the August–December 2021 time period compared to the same age group surveyed in the August–December 2019 time period (OR: 1.21, 95% CI: 1.06–1.38; p = 0.006).

**Table 3:** Odds of past 30-day e-cigarette use late in the COVID-19 pandemic (August–December 2021) compared to prior to the pandemic (August–December 2019) among youth and young adults in the United States, age-adjusted or stratified by age group.

	All ages (15–24 years)	Ages 15–17	Ages 18–20	Ages 21–24
	n = 10,799	n = 2,811	n = 3,392	n = 4,497
	OR (95% CI)	OR (95% CI)	OR (95% CI)	OR (95% CI)
Survey period^a^				
Before COVID-19 pandemic	REF	REF	REF	REF
Late COVID-19 pandemic	1.04 (0.95, 1.14)	1.02 (0.82, 1.25)	0.89 (0.76, 1.04)	1.16 (1.01, 1.32)
Age category				
15–17	0.44 (0.39, 0.49)			
18–20	0.88 (0.79, 0.97)			
21–24	REF			
Race/ethnicity				
White, non-Hispanic	REF	REF	REF	REF
Black, non-Hispanic	0.53 (0.46, 0.62)	0.47 (0.32, 0.69)	0.42 (0.32, 0.55)	0.64 (0.52, 0.78)
Hispanic	0.79 (0.71, 0.88)	0.93 (0.73, 1.19)	0.66 (0.54, 0.81)	0.79 (0.67, 0.94)
Other, non-Hispanic	0.68 (0.58, 0.81)	0.64 (0.44, 0.94)	0.64 (0.47, 0.86)	0.78 (0.60, 1.01)
Perceived financial circumstances^b^				
Low	1.30 (1.19, 1.42)	1.90 (1.55, 2.35)	1.43 (1.22, 1.67)	1.09 (0.96, 1.25)
High	REF	REF	REF	REF
Sensation seeking	1.74 (1.64, 1.85)	1.80 (1.57, 2.07)	1.80 (1.62, 2.01)	1.68 (1.54, 1.84)
Sex				
Male	1.06 (0.97, 1.15)	0.90 (0.74, 1.10)	1.01 (0.86,1.17)	1.16 (1.02, 1.32)
Female	REF	REF	REF	REF
State of residence				
Alaska	0.85 (0.37, 1.96)	3.01 (0.83, 10.88)	0.27 (0.05, 1.48)	0.50 (0.10, 2.41)
Arizona	0.98 (0.65, 1.50)	0.69 (0.25, 1.92)	0.72 (0.34, 1.54)	1.33 (0.73, 2.42)
Arkansas	0.72 (0.44, 1.16)	0.71 (0.17, 2.90)	0.29 (0.11, 0.81)	1.08 (0.57, 2.02)
California	0.88 (0.64, 1.24)	0.81 (0.37, 1.77)	0.69 (0.39, 1.22)	1.06 (0.65, 1.72)
Colorado	1.05 (0.67, 1.63)	0.94 (0.35, 2.57)	1.29 (0.61, 2.74)	0.93 (0.48, 1.80)
Connecticut	1.23 (0.74, 2.04)	1.78 (0.56, 5.65)	1.15 (0.51, 2.58)	1.10 (0.51, 2.36)
Delaware	0.71 (0.29, 1.72)		0.46 (0.12, 1.71)	1.46 (0.36, 5.86)
District of Columbia	0.76 (0.26, 2.16)		0.34 (0.04, 3.09)	1.43 (0.37, 5.46)
Florida	0.87 (0.62, 1.22)	0.63 (0.28, 1.41)	0.77 (0.43, 1.38)	1.06 (0.65, 1.74)
Georgia	0.93 (0.64, 1.34)	0.86 (0.36, 2.04)	0.70 (0.37, 1.33)	1.15 (0.67, 1.97)
Hawaii	1.25 (0.54, 2.87)	1.12 (0.18, 7.10)	1.48 (0.37, 5.87)	0.98 (0.26, 3.61)
Idaho	0.71 (0.34, 1.50)		0.59 (0.18, 1.87)	1.51 (0.46, 4.96)
Illinois	0.98 (0.67, 1.44)	0.85 (0.36, 2.03)	0.80 (0.42, 1.53)	1.19 (0.67, 2.09)
Indiana	0.87 (0.58, 1.32)	1.01 (0.39, 2.63)	0.44 (0.21, 0.92)	1.29 (0.72, 2.34)
Iowa	0.70 (0.40, 1.22)	0.65 (0.15, 2.87)	0.69 (0.30, 1.57)	0.65 (0.26, 1.61)
Kansas	0.87 (0.50, 1.52)	1.35 (0.47, 3.87)	0.34 (0.11, 1.02)	1.17 (0.51, 2.69)
Kentucky	1.31 (0.85, 2.00)	1.42 (0.53, 3.84)	1.21 (0.59, 2.48)	1.35 (0.72, 2.53)
Louisiana	1.17 (0.76, 1.81)	1.50 (0.55, 4.01)	1.12 (0.55, 2.28)	1.15 (0.61, 2.16)
Maine	0.40 (0.16, 0.99)		0.50 (0.13, 1.99)	0.46 (0.13, 1.67)
Maryland	0.99 (0.63, 1.55)	0.87 (0.30, 2.47)	0.82 (0.37, 1.80)	1.16 (0.62, 2.19)
Massachusetts	1.40 (0.90, 2.19)	1.56 (0.62, 3.90)	1.12 (0.50, 2.51)	1.51 (0.78, 2.90)
Michigan	1.00 (0.68, 1.49)	1.13 (0.47, 2.74)	0.98 (0.50, 1.91)	0.94 (0.52, 1.69)
Minnesota	1.23 (0.78, 1.95)	0.72 (0.26, 2.02)	1.58 (0.73, 3.42)	1.28 (0.62, 2.63)
Mississippi	1.39 (0.83, 2.33)	2.26 (0.70, 7.35)	0.85 (0.33, 2.19)	1.57 (0.77, 3.21)
Missouri	0.88 (0.57, 1.37)	0.82 (0.31, 2.14)	1.24 (0.60, 2.56)	0.60 (0.29, 1.21)
Montana	0.96 (0.34, 2.68)	1.29 (0.09, 18.99)		1.84 (0.49, 6.86)
Nebraska	2.37 (1.32, 4.26)	0.38 (0.07, 1.94)	1.08 (0.37, 3.15)	5.91 (2.46, 14.18)
Nevada	1.30 (0.74, 2.28)	1.72 (0.62, 4.76)	0.51 (0.15, 1.75)	1.66 (0.71, 3.89)
New Hampshire	1.49 (0.73, 3.05)		2.23 (0.61, 8.19)	1.99 (0.67, 5.88)
New Jersey	0.95 (0.63, 1.45)	0.88 (0.34, 2.30)	1.17 (0.57, 2.40)	0.89 (0.48, 1.64)
New Mexico	1.45 (0.68, 3.06)	2.35 (0.55, 10.10)	1.94 (0.39, 9.66)	1.07 (0.38, 3.01)
New York	1.01 (0.71, 1.42)	1.19 (0.53, 2.67)	0.80 (0.44, 1.45)	1.09 (0.66, 1.79)
North Carolina	1.14 (0.78, 1.67)	1.52 (0.65, 3.56)	0.78 (0.41, 1.48)	1.28 (0.73, 2.25)
North Dakota	1.16 (0.35, 3.80)		5.20 (0.76, 35.52)	0.29 (0.03, 2.96)
Ohio	0.70 (0.48, 1.02)	0.59 (0.24, 1.46)	0.56 (0.29, 1.08)	0.85 (0.50, 1.47)
Oklahoma	0.88 (0.55, 1.42)	0.40 (0.09, 1.67)	0.49 (0.20, 1.18)	1.52 (0.79, 2.93)
Oregon	1.19 (0.72, 1.96)	0.44 (0.11, 1.74)	1.51 (0.62, 3.65)	1.39 (0.66, 2.91)
Pennsylvania	1.05 (0.73, 1.53)	1.20 (0.52, 2.80)	1.12 (0.61, 2.06)	0.84 (0.47, 1.49)
Rhode Island	0.71 (0.28, 1.80)	1.36 (0.14, 13.70)	0.69 (0.19, 2.49)	0.50 (0.10, 2.53)
South Carolina	1.17 (0.76, 1.81)	1.82 (0.69, 4.85)	1.06 (0.50, 2.20)	1.05 (0.56, 1.98)
South Dakota	1.48 (0.60, 3.68)	2.83 (0.68, 11.82)		3.78 (1.04, 13.75)
Tennessee	1.05 (0.71, 1.55)	0.94 (0.36, 2.48)	1.21 (0.62, 2.35)	0.95 (0.53, 1.68)
Texas	1.06 (0.76, 1.48)	0.64 (0.28, 1.45)	0.84 (0.48, 1.50)	1.51 (0.93, 2.46)
Utah	1.05 (0.63, 1.76)		1.05 (0.44, 2.53)	1.69 (0.79, 3.63)
Vermont	1.33 (0.49, 3.56)		0.96 (0.19, 4.84)	2.14 (0.53, 8.62)
Virginia	0.90 (0.60, 1.35)	0.50 (0.17, 1.45)	0.89 (0.45, 1.77)	1.05 (0.59, 1.91)
Washington	1.20 (0.77, 1.86)	1.47 (0.58, 3.75)	0.69 (0.32, 1.49)	1.60 (0.83, 3.09)
West Virginia	1.14 (0.63, 2.07)	1.90 (0.41, 8.75)	0.74 (0.29, 1.86)	1.35 (0.55, 3.32)
Wisconsin	1.06 (0.66, 1.69)	0.83 (0.28, 2.44)	0.72 (0.34, 1.53)	1.67 (0.79, 3.54)
Wyoming	0.41 (0.09, 1.74)			1.06 (0.21, 5.29)

^a^Respondents surveyed August–December 2019 were classified in the “Pre-COVID-19 pandemic” category, and those surveyed August–December 2021 were classified in the “Late COVID-19 pandemic” category.

^b^Lower perceived financial circumstances do not meet basic needs or just met needs with nothing left over. Higher perceived financial circumstances meet basic needs with a little left over or allow the respondent to live comfortably.

## Discussion

The findings of this study indicate that e-cigarette use has returned to pre-COVID-19 prevalence rates among young people aged 15–24 years old in the United States. Among young adults aged 21–24 years, evidence indicates that the likelihood of e-cigarette use is significantly higher as compared to the period before the pandemic. In 2019, immediately prior to the COVID-19 pandemic, e-cigarette use had reached unprecedented rates of use among young people in the United States [3, 4].

These findings are observed as young people increase their engagement in social activities and attendance for in-person schooling with the progression of the COVID-19 pandemic in the United States. While previous research indicated that younger people reported reduced e-cigarette access due to COVID 19 restrictions, young adults did not experience the same access issues [6]. As adolescents and college-enrolled young adults return to school, they once again encounter peer e-cigarette use, which increases accessibility and can serve to normalize vape use behaviors. Other research has found that young e-cigarette users report increasing use to cope with stress [7]; those emerging from the pandemic may encounter new stressors following several years of remote learning and working. Furthermore, e-cigarette manufacturers have continued to market products throughout the pandemic, including messages with youth appeal and/or specific to COVID-19 (e.g., health reassurances) [12]. Exposure to e-cigarette marketing has been shown to be a factor in initiation and progression to regular use of these products among young people [13–17].

Evidence is emerging on the effectiveness of anti-vaping interventions that denormalize e-cigarette use among young people, contributing to a reduced risk of initiation and increased likelihood of quitting [18, 19]. Greater opportunities for social contacts among adolescents and young adults are to be expected as society continues to adapt to and recover from the COVID-19 pandemic, and with them the increased potential to obtain e-cigarettes from social contacts and observe e-cigarette use behaviors among peers. Health communication efforts targeting adolescents and young adults that portray e-cigarettes as socially unacceptable among important others, including peers, is increasingly relevant and critical.

Schools and colleges can take steps to reduce e-cigarette use on campus through policy implementation, though the presence of a policy alone may not be sufficient [20]. Schools need to train staff to be aware of emerging products, and how to intervene in non-punitive ways to reduce underage access and use [21]. Additionally, flavor policy restrictions at the local level can play a role in reducing retail access to flavored e-cigarettes, thus reducing the likelihood of underage use [22]. Taken together, these policies both in the school environment and at the local level can limit underage access to e-cigarettes.

### Limitations

Despite this study’s many strengths, there are several limitations. First, despite sampling quotas and survey weights applied to the data, the survey was conducted among a convenience sample and cannot be considered nationally representative. Estimates of the prevalence of e-cigarette use may therefore be biased to an unknown extent and are not directly comparable to nationally representative samples surveyed using other methods (e.g., classroom-based surveys). However, the continuous tracking methodology and large sample size strengthen the study’s internal validity and allow precision when comparing segments of the study population surveyed during different points in time. Second, the survey did not include a measure of respondents’ sources for e-cigarettes prior to the COVID-19 pandemic, limiting conclusions about the relative role of retail and social sources played in adolescents and young adult access to e-cigarette products before, compared to during, the pandemic. Future research comparing information on sources of e-cigarettes pre- and post-COVID-19 can help create relevant policy interventions to best reduce access. Third, although research suggests that youth and young adults were using e-cigarettes to cope with stress during the COVID-19 pandemic [7], the study did not control for respondents’ mental health status due to the lack of comparable measures of mental health on the survey over time. We therefore cannot use the study findings to draw conclusions about the role of mental health in the observed greater likelihood of e-cigarette use later in the pandemic. The interaction between the COVID-19 pandemic, youth and young adult mental health, and trajectories of nicotine and other substance use warrants further research, ideally using longitudinal samples followed prior to and beyond the COVID-19 pandemic.

## Conclusions

Renewed attention must be paid to the re-emerging epidemic of e-cigarette use among young people. The temporary decline in e-cigarette use during the COVID-19 pandemic [6], followed by the increase observed in the current study, is indicative of the powerful role of social influences and points of access on e-cigarette use among adolescents and young adults. Evidence-based tobacco control efforts are urgently needed, including further restrictions on sales of e-cigarettes with youth appeal (e.g., flavored products), as well as targeted public health campaigns and cessation services, to encourage the next generation to reject tobacco, vaping and nicotine.
